# ATF1/miR-214-5p/ITGA7 axis promotes osteoclastogenesis to alter OVX-induced bone absorption

**DOI:** 10.1186/s10020-022-00476-7

**Published:** 2022-05-14

**Authors:** Lu-Lin Liu, Yao-Sheng Xiao, Wei-Min Huang, Sheng Liu, Li-Xing Huang, Jing-Hua Zhong, Peng Jia, Wu-Yang Liu

**Affiliations:** 1 Department of Orthopedics, The First Affiliated Hospital of Gannan Medical University, No.128, Jinling Road, Ganzhou, 341000 Jiangxi People’s Republic of China; 2 Department of Gastroenterology, The First Affiliated Hospital of Gannan Medical University, Ganzhou, 341000 Jiangxi People’s Republic of China; 3 Department of Oncology, The First Affiliated Hospital of Gannan Medical University, Ganzhou, 341000 Jiangxi People’s Republic of China; 4 Department of Orthopedics, Second Affiliated Hospital of Soochow University, Suzhou, 215004 Jiangsu People’s Republic of China

**Keywords:** ATF1, ITGA7, miR-214-5p, Osteoclastogenesis, Osteoporosis

## Abstract

**Background:**

The dynamic balance of osteoblast and osteoclast is critical for bone homeostasis and overactive osteoclastic function may lead to osteoporosis. Activating transcription factor 1 (ATF1) is involved in osteoclastogenesis. However, the detailed mechanisms remain to be explored.

**Methods:**

RAW264.7 cells were used and induced toward osteoclast by RANKL administration. We performed flow cytometry, CCK-8 assay and tartrate-resistant acid phosphatase (TRAP) staining to examine cell apoptosis, proliferation and differentiation of RAW264.7 cells, respectively. Mice were subjected to ovariectomy to induce osteoporosis. Micro CT, HE staining and TRAP staining were performed to evaluate bone loss in the OVX mouse model. Bioinformatics methods, luciferase assays and Chromatin Immunoprecipitation (ChIP) were used to predict and validate the interaction among ATF1, miR-214-5p, and ITGA7.

**Results:**

ATF1 and miR-214-5p were up-regulated while ITGA7 was inhibited in RANKL-induced osteoclasts. MiR-214-5p was transcriptionally activated by ATF1. ATF1 knockdown suppressed osteoclast formation by miR-214-5p inhibition. ITGA7 was the direct target of miR-214-5p. Knockdown of miR-214-5p abolished osteoclastogenesis, which was reversed by ITGA7 knockdown. In OVX model, miR-214-5p knockdown suppressed osteoclast differentiation and prevented bone loss.

**Conclusion:**

ATF1/miR-214-5p/ITGA7 axis regulated osteoclast formation both in vivo and in vitro, thereby affecting OVX-induced bone resorption in mice. Knockdown of ATF1 might be a promising strategy to manage osteoporosis.

**Supplementary Information:**

The online version contains supplementary material available at 10.1186/s10020-022-00476-7.

## Introduction

Osteoporosis is a type of skeletal disease, presenting abnormal bone homeostasis in which bone resorption overpowered bone formation (Harslof and Langdahl 2016; Raisz 2005). Usually, the elderly have high risk of osteoporosis, especially in postmenopausal women, and life qualities will be severely deteriorated (Pawlowski et al. 2015). As is widely investigated, the balance of bone formation and resorption is critical for bone homeostasis (Alliston and Derynck 2002). Therefore, elucidation of mechanisms involved in this balance is critical for osteoporosis treatment.

Osteoclasts are the main functional cells of bone resorption and play important roles in bone development, growth, repair, and reconstruction (Jacome-Galarza et al. 2019). Deficiency of osteoclast activity leads to bone sclerosis and bone marrow failure, while excessive activity can lead to bone loss and osteoporosis (Yao et al. 2017). Therefore, an exploration of the mechanisms involved in osteoclastogenesis will be helpful in the development of therapy against osteoporosis.

ATF1 has a unique molecular structure and biological functions as fusion or non-fusion gene in the occurrence and development of tumors (Komura et al. 2019). In osteoclastogenesis, Kazuki identified ATF1 as a novel transcription factor by DNase-seq (Inoue and Imai 2014). Specifically, ATF1 expression was moderately elevated in osteoclast differentiation, and ATF1 down-regulation reduced TRAP-positive osteoclasts after RANKL treatment. Nonetheless, the relevant mechanisms were not further investigated. Multiple miRNAs participate in the regulation of osteoporosis by modulating osteoclastogenesis (Li et al. 2019; Li et al. 2018; Zhao et al. 2017). MiR-214-5p was induced in patients with glucocorticoid-induced osteoporosis (Ren et al. 2019). Qiu J et al. reported that miR‑214‑5p might disrupt osteogenic differentiation of BMSCs by modulating COL4A1 and aggravate the symptoms of postmenopausal osteoporosis (Qiu et al. 2018). Bioinformatics prediction by JASPAR predicted possible binding sites in the promoter of miR-214-5p for transcription factor ATF1. However, no detailed studies have been performed to manifest the interaction between ATF1 and miR-214-5p.

Integrin subunit alpha 7 (ITGA7) is a member of integrin alpha family and mediates cell adhesion (Yao et al. 1996). In the milieu of osteoporosis, Huang T and colleagues demonstrated that ITGA7 was drastically inhibited in ovariectomy (OVX) mice. Overexpression of ITGA7 improved osteogenesis via phosphatidylinositol 3-kinase/Akt (PI3K/Akt) pathway (Huang et al. 2020). Moreover, ITGA7 was predicted to be the target of miR-214-5p by Targetscan. Whereas the interaction between miR-214-5p and ITGA7 has not been reported previously.

Based on the relevant studies and bioinformatics prediction, this study hypothesizes that transcription factor ATF1 will activate miR-214-5p, which then inhibits the expression of ITGA7 specifically, thereby promoting osteoclastogenesis and altering OVX-induced bone resorption in mice.

## Materials and methods

### Cell culture

RAW264.7 cells were from the Shanghai Cell Collection (Chinese Academy of Sciences, Shanghai, China). Cells were cultured using Dulbecco Modified Eagle Medium (DMEM, Gibco, Carlsbad, CA, USA), in addition with 10% fetal bovine serum (FBS, Gibco, Carlsbad, CA, USA), 1% penicillin–streptomycin solution (Thermo Fisher Scientific, MA, USA). All cell lines were cultured under 5% CO_2_, with a temperature of 37 °C. To induce osteoclastogenesis, RAW264.7 cells were incubated with 50 ng/ml of RANKL (Thermo Fisher Scientific) for 3–5 days (Wang et al. 2020).

### BMSCs isolation and osteogenesis induction

To separate BMSC from the medullary cavity, female mice were sacrificed, and BMSC were washed from the femora and treated at 4 °C for 20 min with FITC, PE, and allophycocyanin conjugated antibodies, as well as peridinin chlorophyll protein conjugated to CD29, CD45, CD11b, and Sca1 (BioLegend). Acquisition and analysis were carried out using the FACS Aria model and FACS DIVE software version 6.1.3 (BD Biosciences). Primary BMSC were isolated and planted in culture flasks for cell population enrichment. They were subcultured about 1 week later, when the secondpassage BMSC clustered. The osteogenesis induction media (5 mmol/L glycerophosphate and 50 g/mL ascorbic acid, 300 ng/mL BMP2) was applied to BMSC for 48 h in 24-well plates (5 × 10^5^ cells/well). Then, homogenize the cell lysates to determine ALP activity using the enzymatic colorimetric ALP Kit (from Roche) and spectrophotometric determination of the output of pnitrophenol. The amounts of secreted osteocalcin were determined in culture media using an immunoassay kit (DiaSorin).

### Primary osteoclasts isolation

To isolate adult mouse osteoclast precursors, complete bone marrow is extracted from 8 to 12-week-old mice's long bones (tibia and femur). To begin, scissors are used to remove the deceased mice's hindlimbs while leaving the lengthy bones intact. The limbs' paws and skin are separated and packed in PBS for shipping. Transfer the limbs to a sterile Petri dish and cut through the knee joint with a scalpel to separate the femur and tibia. The remaining soft tissue on the bones is scraped away, and the epiphyses are severed to reveal the bone marrow. Using a 5 ml syringe fitted with a 25-gauge (G) needle, flush the bone marrow from each bone onto a new sterile 60 mm Petri plate. By passing the cleansed bone marrow through needles of decreasing size (19G2125G) and putting it into a conical 15 ml tube, a single cell suspension is formed. Centrifuge the cell suspension at 300*g* for 3 min, remove the supernatant, and resuspend the pellet in 1 ml of growth media.

### Cell transfection

Short hairpin RNA (shRNA) targeting ATF1, ITGA7 and respective negative controls (NC) were obtained by Shanghai GenePharma (Shanghai, China). The plasmids containing ATF1, miR-214-5p mimic/inhibitor and respective NC were from Genepharma (Suzhou, China). Lipofectamine 3000 reagent (Life Technologies Corporation, Carlsbad, CA, USA) was used for cell transfection.

### RNA extraction and qRT-PCR

TRIzol® reagent (Thermo Fisher Scientific, MA, USA) was utilized to obtain total RNA from tissues and cell lines. NanoDrop™ 2000 Spectrophotometers (Thermo Fisher Scientific, MA, USA) was utilized to test RNA quality. Then the RNA was used to synthesize cDNA by PrimeScript™ RT reagent Kit (Takara, Dalian, China). qRT-PCR was used to determine the genes expression of ATF1, miR-214-5p and ITGA7 using SYBR® Green Real-Time PCR master mix (Thermo Fisher Scientific, MA, USA) on ABI StepOnePlusTM Real-Time PCR System (Applied Biosystems, CA, USA). Each sample was assessed 3 times. GAPDH and U6 were used as reference genes and primer sequence of tested genes were listed below:

ATF1: forward 5′-GAGCAGCGGACAGTACATTG-3′,

ATF1: reverse 5′-CGGATCTGGTAGGTCTGCAT-3′;

miR-214-5p: forward 5′-GCGTGCCTGTCTACACTTG-3′,

miR-214-5p: reverse 5′- GTCGTATCCAGTGCAGGGTCCGAGGTATTCGCACTGGATACGACGCACAG-3′;

ITGA7: forward 5′-GCTGTGAAGTCCCTGGAAGT GATT-3′,

ITGA7: reverse 5′-GCATCTCGGAGCATCAAGTTC TT-3′;

U6: forward 5′-CTCGCTTCGGCAGCACA-3′,

U6: reverse 5′-AACGCTTCACGAATTTGCGT-3′;

GAPDH: forward 5′-CTGACTTCAACAGCGACACC-3′,

GAPDH: reverse 5′-GTGGTCCAGGGGTCTTACTC-3′;

### Western blotting analysis

Cells and tissues were harvested and lysed in RIPA buffer with protease inhibitors (Beyotime Institute of Biotechnology, Shanghai, China) to obtain total protein. After electrophoresis, proteins were transferred to a PVDF membrane (Invitrogen, Grand Island, NY, USA). Treated with 5% skim milk powder, the membrane was incubated with primary antibody including ITGA7 (1:1000), CTSK (1:1000) (Abcam, Cambridge, UK), TRAP (1:500), NFATc1 (1:500), V-ATPase-d2 (1:1000) (Santa Cruz Biotechnology, Heidelberg, Germany) and GAPDH (1:1000, Cell Signaling Technology, MA, USA) at 4 °C overnight. Next, the membrane was washed using TBST. Then membrane was cultured with HRP-conjugated secondary antibody (1:2000, Abcam) at 1 h. After washing, the bands were imaged using Gel Imaging System (Life Science, CA, USA), and the quantification of proteins was examined by ImageJ V1.8.0.112.

### Cell viability assay

Cell proliferation was detected through CCK-8 assay (Beyotime Institute of Biotechnology). Cells were put in a 96-well plate and cultured for 48 h at 37 °C, and then add 10 μl CCK-8 and incubate for 2 h at 37 °C, measure the absorbance at 450 nm at 48 h.

### Apoptosis assay

After collection, cells were stained with Annexin V-PI (Beyotime Institute of Biotechnology) based on instructions and then sorted by flow cytometry (FACScan, BD Bioscience, N.J., USA). Results were evaluated by CELL Quest 3.0 software (BD Bioscience, N.J., USA). When Annexin V was used in combination with PI, PI was excluded from viable cells (FITC-/PI-) and early apoptotic cells (FITC + /PI-), while late apoptotic cells and necrotic cells were both positive for FITC and PI.

### ChIP assays

JASPAR database was utilized to predict potential interaction between miR-214-5p promoter and ATF1. SimpleChIP® Kit (Cell Signaling Technology) was utilized for ChIP assay. Briefly, RAW264.7 cells were harvested and treated with 1% formaldehyde for 10 min. After crosslink, the chromatin was lysed and broken to fragments ranged from 200 to 1000 bp by ultralsound. Chromatin was then immunoprecipitated with 2 μg of ATF1 or IgG antibody (Abcam) at 4 °C for 2 h. The product was examined by qRT-PCR. The experiments were repeated for 3 times. Primer sequences: site-1 (F) 5′-AGGGAGAGGGGAAAGCAATA-3′, (R) 5′-ACGTGTGCTTCTGTCCAACA-3′; site-2 (F) 5′-GAGGGCCAGTAACAACAGGA-3′, (R) 5′-GACAGGGGGATAAAGGGAAA- 3′.

### Alizarin Red Staining

After 7 or 14 days of cordycepin treatment, cells were fixed for 10 min at room temperature in 4% paraformaldehyde. Fixed cells were rinsed three times with distilled water and then stained for 10 min at room temperature with alizarin red s solution. To quantify the cells, they were dissolved in PBS and transferred to 96 well plates, where their absorbance at 550 nm was quantified using a microplate reader.

### Dual-luciferase reporter assays

Targetscan was utilized to predict potential binding sites between miR-214-5p and 3′ UTR of ITGA7. The binding site was mutated to construct an ITGA7 mutant (ITGA7-MUT) by a rapid site-directed mutagenesis kit (KM101, TIANGEN, Beijing). The predicted binding sites of miR-214-5p on ITGA7 (ITGA7-WT) and its mutated site (ITGA7-MUT) were constructed into the pmirGLO vector (Promega, Madison, WI, USA). RAW264.7 cells were co-transfected with ITGA7-WT/MUT constructs and miR-214-5p mimic/mimic NC. After 48 h, examine luciferase activities with the Dual-Glo® Luciferase Assay System (Promega). For miR-214-5p promoter luciferase assay, the miR-214-5p promoter binding site was mutated and constructed by a rapid site-directed mutagenesis kit (KM101, TIANGEN, Beijing). The miR-214-5p-WT and-MUT (site 1–2) promoter was integrated into pGL3-Basic (Promega). pGL3-miR-214-5p promoter and sh ATF1 or ATF1, as well as the respective control (sh NC or vector) were transfected into cells. Dual-luciferase assay was conducted for three times.

### Mouse ovariectomized model

All animal experiments were approved by the Institutional Animal Care and Use Committee of Soochow University. 3-month-old C57BL/6 female mice were designated to 4 groups: ovariectomized group (OVX, n = 6), sham group (Sham, n = 6), OVX + antagomir NC (n = 6) and OVX + miR-214-5p antagomir (n = 6). OVX group was subjected to bilateral ovariectomy 7 days after acclimatization, while intact ovaries were retained in sham group. To prevent infection, each mouse was injected penicillin within the 3 days after operation. 100 μl antagomir NC and miR-214-5p antagomir were given through tail vein injection and the concentration was 1*10^9^ TU/ml after ovariectomy. After 4 weeks, tibia bone tissues were harvested for subsequent assays (Zhou et al. 2019).

### Micro-CT scanning

Micro-CT80 system (Scanco Medical, Switzerland) was used to evaluate the bone status. Scan was performed under a parameter of 70 kV and 70 mA X-ray energy, 10 mm equidistant definition, and a voxel size of 10 mm. The region of interest was picked for further analysis. Bone mineral density (BMD), trabecular number (Tb.N) and trabecular thickness (Tb.Th) was examined to investigate trabecular structure.

### Hematoxylin and eosin (H&E) staining

For HE staining, tissues were treated with 4% paraformaldehyde and embedded in paraffin based on the HE protocol. Then the slides were deparaffinized in dimethylbenzene followed by administration of hematoxylin (Sigma-Aldrich, St.Louis, MO, USA) for 5 min and eosin (Sigma-Aldrich) for 2 min. Then the slides were rinsed with water for 2 times and observed under microscope (Carl Zeiss, Oberkochen, Germany).

### TRAP staining

TRAP staining was conducted on paraffin sections or cultured cells. For paraffin sections, trap staining was conducted as previously described (Solberg et al. 2014). For cells, after differentiation toward osteoclast, cells were treated with 4% paraformaldehyde for 15 min. After washing, cells were exposed to the mixture of acetone and ethanol at a ratio of 1:1. After 30 s treatment, cells were treated with 0.01% naphthol AS-MX phosphate and 0.06% Fast Red Violet LB Salt (387A, Sigma-Aldrich, St.Louis, MO, USA). Staining results were observed under microscope. Mature osteoclasts were TRAP-positive multinucleated cells (> 3 nuclei/cell) and the number was counted.

The TRSAP-positive multinucleated cells attached to the bone surface were counted as osteoclasts, and parameters such as the number of osteoclasts per bone surface (N.Oc/BS) and the number of osteoclasts per bone surface (Oc.S/BS) were obtained.

### Statistical Analysis

Experiments were conducted more than 3 times. Data was presented as the mean ± standard deviation (SD). SPSS 22.0 was used to analyze data. Unpaired two-tailed Students’ t-test was used for comparison of two groups, One-way analysis of variance (ANOVA) followed by Tukey post hoc test was utilized for multiple groups. The statistical significance was P < 0.05.

## Results

### ATF1 and miR-214-5p are overexpressed and ITGA7 is downregulated in RANKL induced osteoclast

**Fig. 1:**
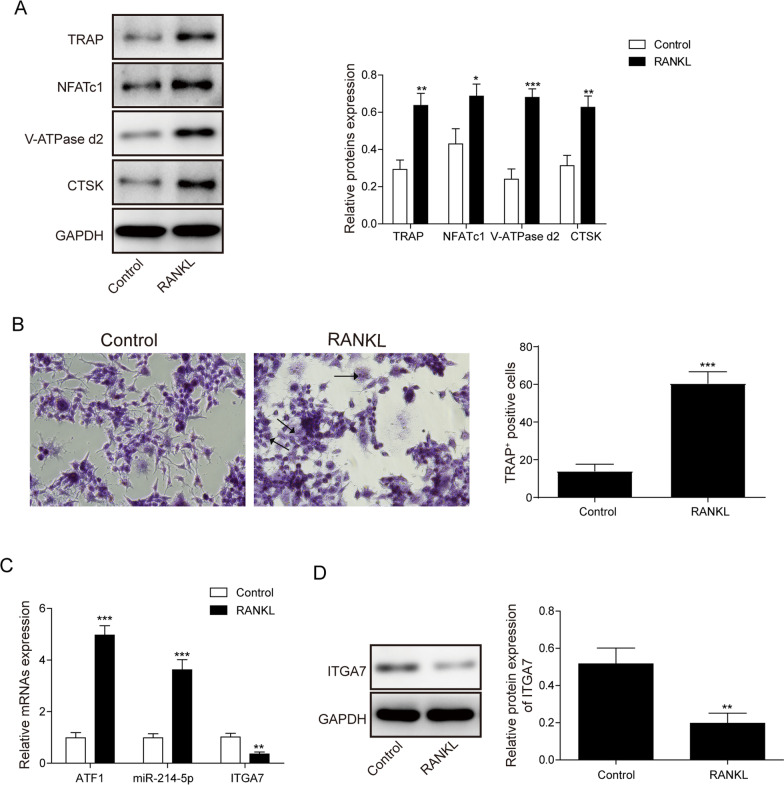
ATF1, miR-214-5p are upregulated and ITGA7 is downregulated in RANKL-induced osteoclast. **A** Expression of osteoclast markers examined by western blotting. **B** TRAP staining results in control and RANKL treatment group. **C** Expression of ATF1, miR-214-5p and ITGA7 examined by qRT-PCR. **D** Protein expression of ITGA7 examined by western blotting. Experiments were performed for at least 3 times. Data are shown as mean ± SD. *P < 0.05, **P < 0.01 and ***P < 0.001.

RANKL was widely used to induce osteoclast differentiation (Park et al. 2017). In our research, we examined the efficiency of differentiation via western blotting and TRAP staining after RANKL treatment. In the treatment group, the biomarkers of osteoclast, such as TRAP, NFATc1, V-ATPase-d2 and CTSK were upregulated vs control group (Fig. 1A). Consistently, the number of TRAP positive cells increased significantly in RAW264.7 cells cultured with RANKL (Fig. 1B). Collectively, these results showed high induction efficiency of RANKL. Inoue reported that ATF1 was a novel transcription factor for osteoclastogenesis (Inoue and Imai 2014). In our research, we also examined its expression levels in control and RANKL-treated group. qRT-PCR results indicated an increase in the expression of ATF1 and miR-214-5p in RANKL group, while ITGA7 was suppressed after RANKL treatment (Fig. 1C). Similarly, the protein expression of ITGA7 was also inhibited after osteoclast differentiation (Fig. 1D). Moreover, we validated these results in primary osteoclasts and acquired consistent results (Additional file 2: Fig. S2A–D). Therefore, ATF1 and miR-214-5p were enriched and ITGA7 was suppressed after RANKL treatment.

### ATF1 activates miR-214-5p transcriptionally

**Fig. 2:**
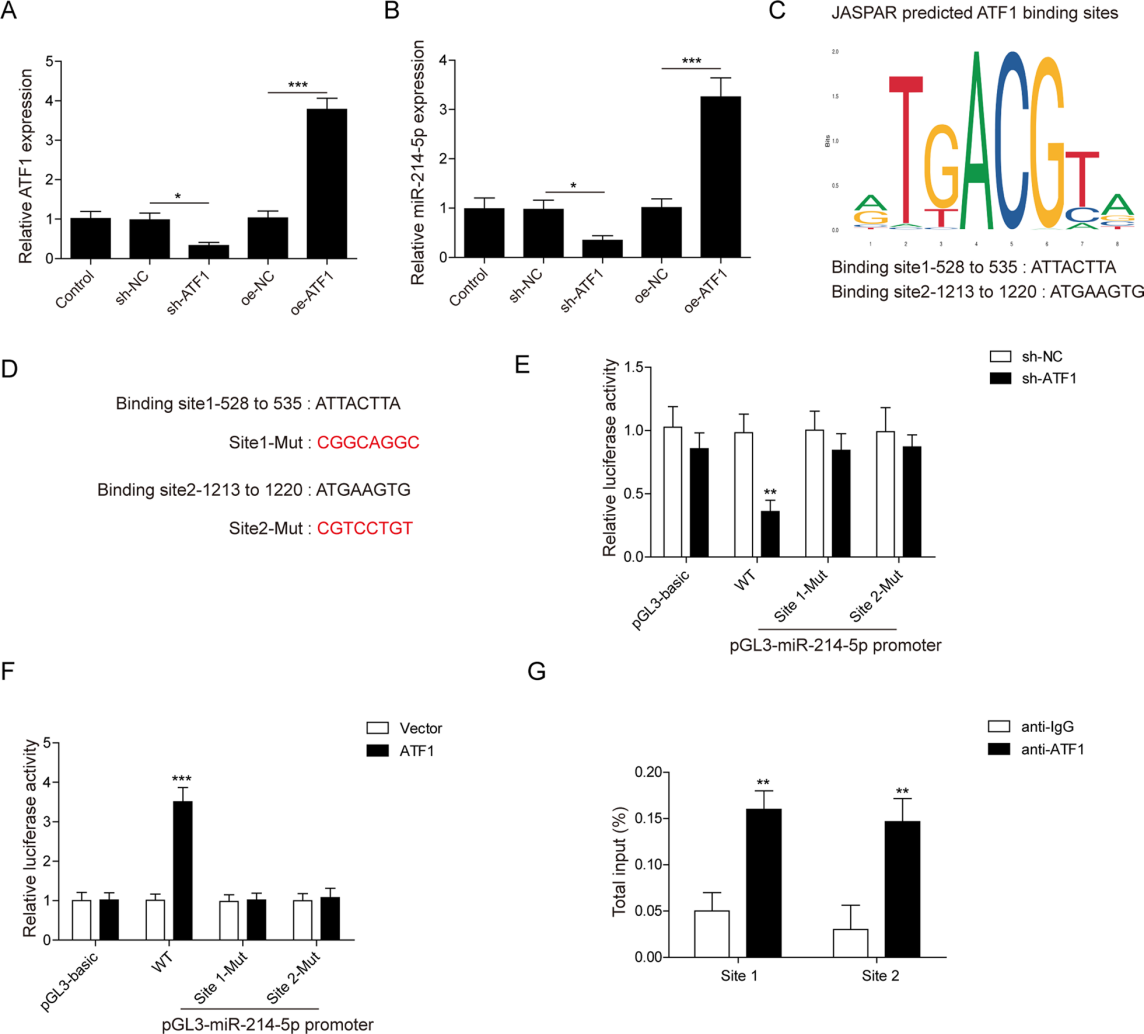
MiR-214-5p is transcriptionally activated by ATF1. Knockdown or overexpression of ATF1 were performed in RAW264.7 cells, ATF1 (**A**) and miR-214-5p (**B**) expression in each group were detected via qRT-PCR. **C** Two predicted binding sites by JASPAR. **D** ATF1 and miR-214-5p promoter binding site mutation sequence. **E**, **F** Luciferase reporter assay in cells transfected with a plasmid containing miR-214-5p-WT, Site 1-MUT and Site 2-MUT. Sh-ATF1 and ATF1 overexpression vector were administrated, respectively. **G** ChIP assays was conducted in RAW264.7 cells. qRT-PCR was utilized to examine miR-214-5p promoter expression associated with ATF1. Experiments were performed for at least 3 times. Data are presented as mean ± SD. *P < 0.05, **P < 0.01 and ***P < 0.001

To figure out the hierarchical relationship, expression of miR-214-5p and ATF1 was manipulated in RAW264.7 cells. The expression of ATF1 was boosted in ATF1 overexpression (oe) group and inhibited in sh ATF1 group (Fig. 2A). Meanwhile, the expression of miR-214-5p also showed the same trend with ATF1 (Fig. 2B). ATF1 was known as a transcription factor, therefore we wondered if ATF1 could bind to the promoter of miR-214-5p. Prediction by JASPAR database pointed out 2 possible binding sites in the promoter of miR-214-5p (Fig. 2C). Luciferase vectors with WT or site1-2 MUT (Fig. 2D) were constructed to investigate whether ATF1 bound to the promoter of miR-214-5p directly. Transfection of sh ATF1 significantly reduced the luciferase activity of WT and site 1–2-MUT transfected cells (Fig. 2E). Conversely, overexpression of ATF1 sharply increased luciferase activity in WT and site 1–2-MUT group (Fig. 2F). To further corroborate this result, ChIP assay was performed and the promoter of miR-214-5p was highly enriched in site 1–2 group (Fig. 2G). To summarize, ATF1 could bind to the promoter of miR-214-5p and activated its expression.

### ATF1 inhibition suppresses osteoclastogenesis in vitro and miR-214-5p reverses this process

**Fig. 3:**
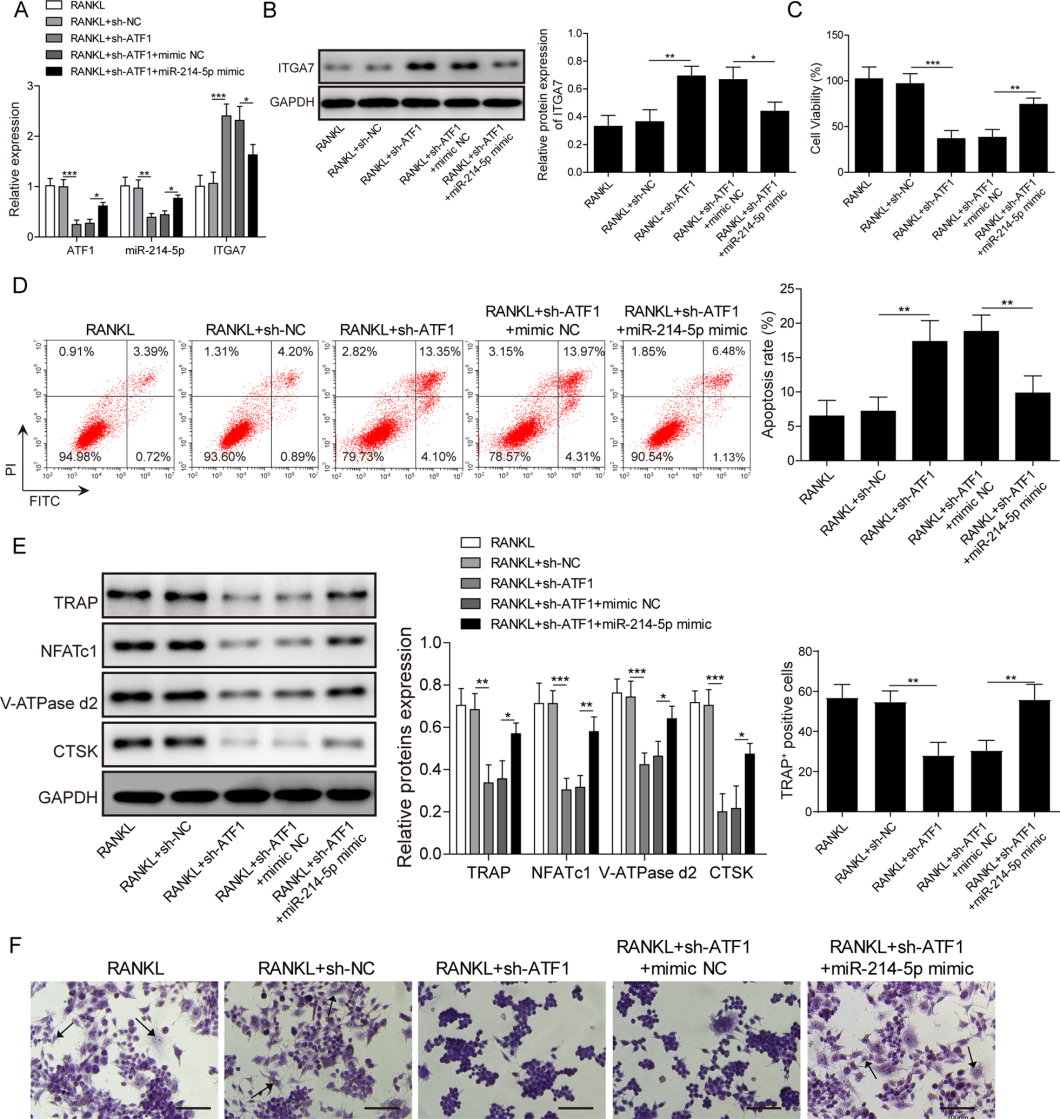
ATF1 inhibition abolishes osteoclast formation, which is reversed by miR-214-5p upregulation. RAW264.7 cells were administrated with sh ATF1/sh NC with or without miR-214-5p mimic/mimic NC. **A** Expression of ATF1, miR-214-5p and ITGA7 was detected by qRT-PCR. **B** Protein level of ITGA7 was detected by western blotting. **C** Cell viability was evaluated by CCK-8 assay. **D** Cell apoptosis was assessed by flow cytometry. **E** Expression of osteoclast markers was evaluated by western blotting. **F** TRAP staining results in each group. Experiments were performed for at least 3 times. Data are presented as mean ± SD. *P < 0.05, **P < 0.01 and ***P < 0.001

The role of ATF1 in the formation of osteoclast was rarely reported in previous studies (Inoue and Imai 2014). We checked out the regulation among ATF1, miR-214-5p and ITGA7. After sh ATF1 treatment, the expression of miR-214-5p was also inhibited (Fig. 3A). While ITGA7 was activated at both mRNA level and protein level. Administration of miR-214-5p mimic inhibited ITGA7 expression (Fig. 3A, B). We then explored the role of ATF1 in osteoclast proliferation and apoptosis. RAW264.7 cells were separated into 4 groups and assigned with the sh ATF1 or sh ATF1 with miR-214-5p mimic or mimic NC. In terms of proliferation, CCK-8 assay revealed that co-treatment of miR-214-5p mimic and sh ATF1 was capable of completely reversing proliferation inhibition caused by sh ATF1 (Fig. 3C). While flow cytometry showed that sh ATF1 treatment enhanced cell apoptosis, which was obviously abrogated by miR-214-5p mimic (Fig. 3D). Differentiation toward osteoclast was also interrupted by sh ATF1, biomarkers of osteoclast like TRAP, NFATc1, V-ATPase-d2 and CTSK were suppressed in sh ATF1 group. Addition of miR-214-5p mimic reversed this trend (Fig. 3E). Similarly, TRAP staining showed that osteoclastogenesis was inhibited by sh ATF1. MiR-214-5p mimic reversed this process (Fig. 3F). All these results demonstrated ATF1 knockdown repressed osteoclastogenesis, which was reversed by miR-214-5p mimic.

### MiR-214-5p directly targets the 3′-UTR of ITGA7

**Fig. 4:**
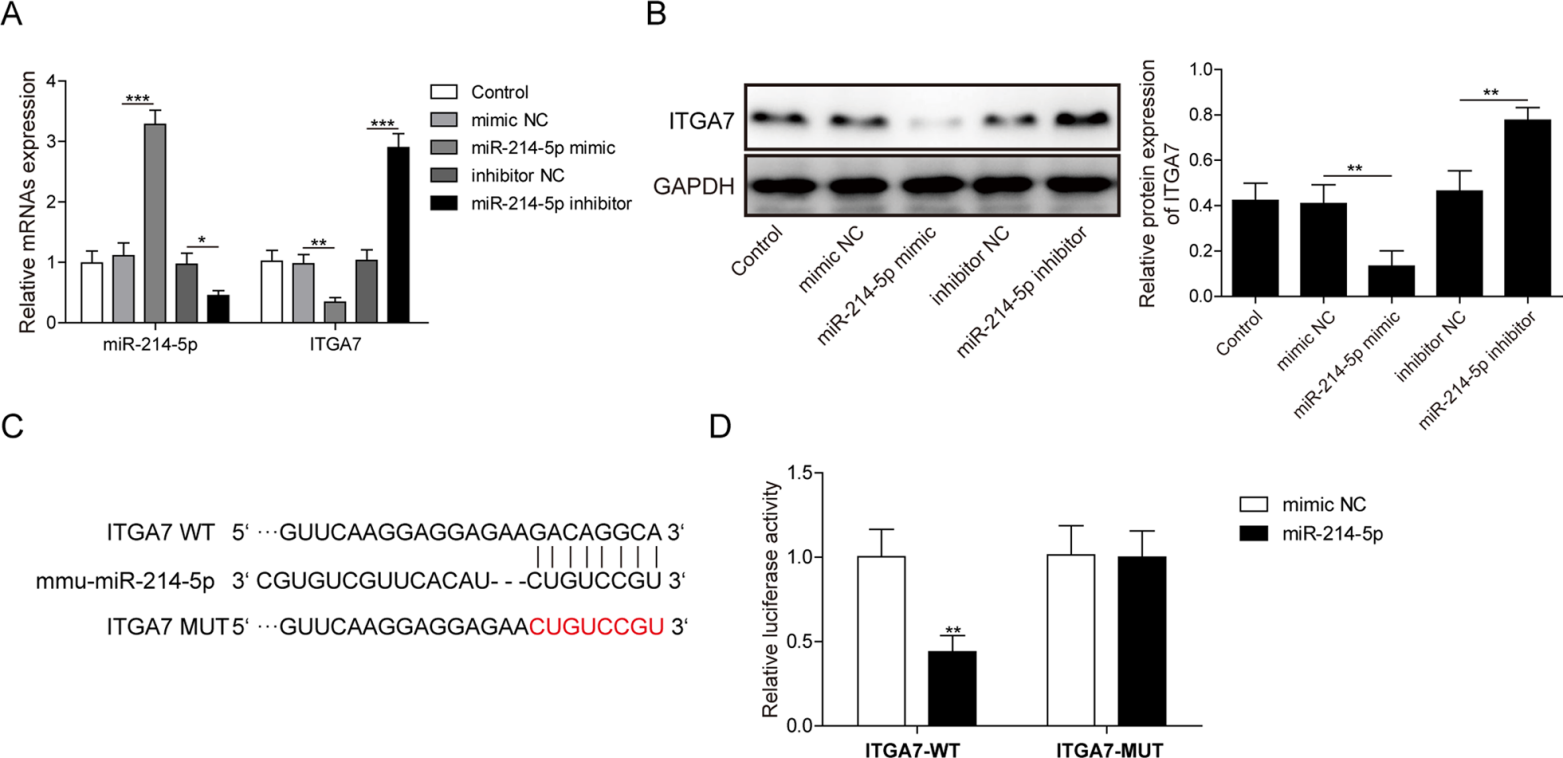
MiR-214-5p directly interacts with ITGA7. Cells were treated with miR-214-5p mimic/mimic NC or inhibitor NC/ miR-214-5p inhibitor. **A** Expression of miR-214-5p and ITGA7 after different treatment was detected by qRT-PCR. **B** Protein level of ITGA7 was detected by western blotting. **C** The possible binding site of miR-214-5p in the ITGA7 3′-UTR. **D** Luciferase reporter assay in RAW264.7 cells transfected with a plasmid containing ITGA7 WT, ITGA7 MUT, miR-214-5p mimic/mimic NC. Experiments were performed for at least 3 times. Data are presented as mean ± SD. *P < 0.05, **P < 0.01 and ***P < 0.001

ITGA7 is a member of integrin family and plays critical roles in osteogenesis (Huang et al. 2020). We detected the interaction between miR-214-5p and ITGA7. The results of qRT-PCR and western blotting revealed that the changes of miR-214-5p could negatively regulate ITGA7 (Fig. 4A, B). Bioinformatics analysis by Targetscan predicted a potential binding site between miR-214-5p and ITGA7 (Fig. 4C). According to dual luciferase assays, activity in ITGA7 WT group was repressed by miR-214-5p transfection in cells. Mutation of binding sites abolished the effects of miR-214-5p mimic transfection (Fig. 4D). All these pointed out that ITGA7 acted as the direct downstream target of miR-214-5p.

### MiR-214-5p regulated ITGA7 and affected bone homeostasis

**Fig. 5:**
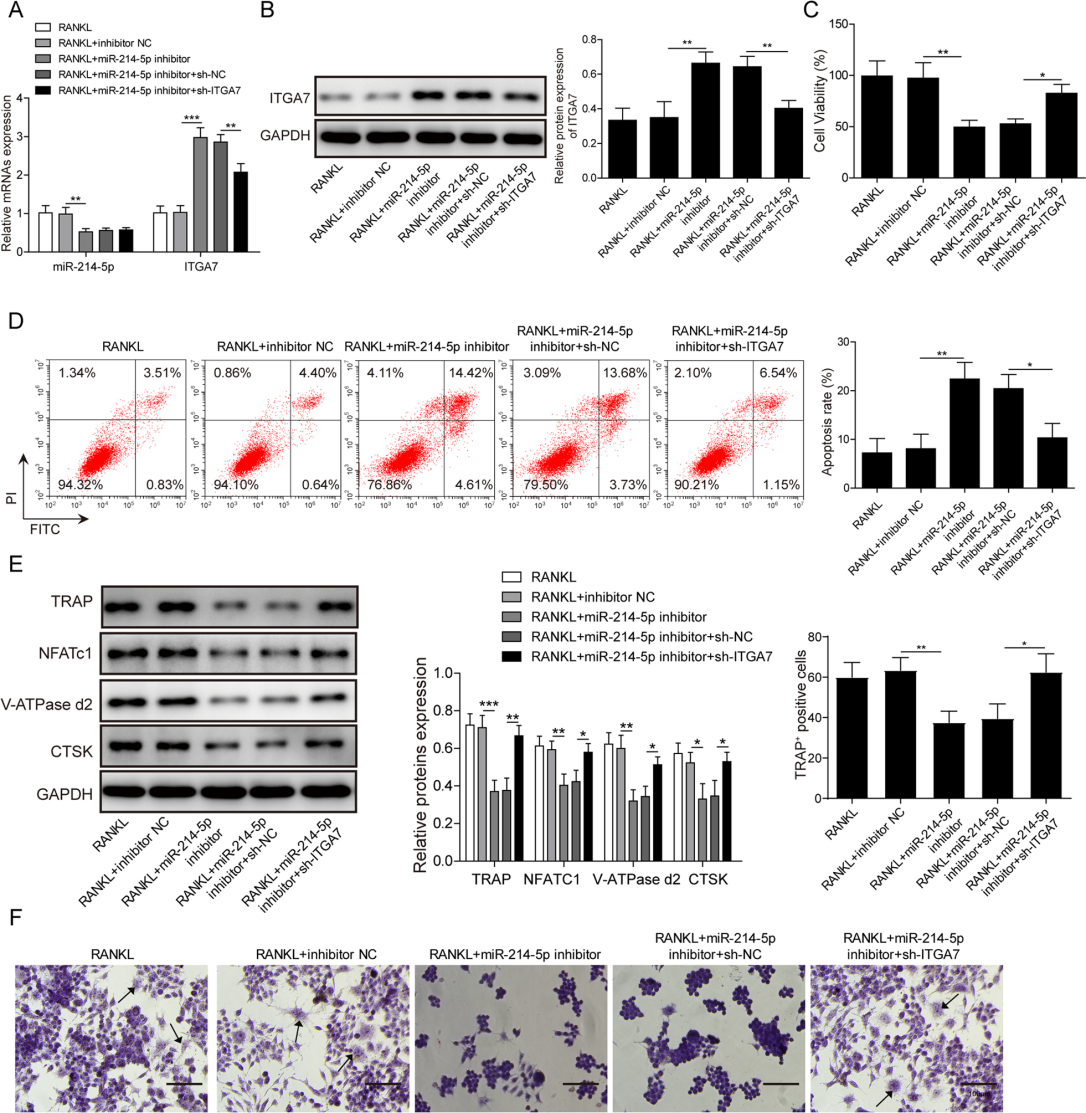
MiR-214-5p downregulation inhibits osteoclastogenesis and ITGA7 knockdown reverses this trend. RAW264.7 cells were treated with miR-214-5p mimic/mimic NC with or without sh ITGA7/sh NC. **A** Expression of miR-214-5p and ITGA7 was examined by qRT-PCR. **B** Protein level of ITGA7 was examined by western blotting. **C** Cell viability was evaluated by CCK-8 assay in each group. **D** Cell apoptosis was assessed by flow cytometry. **E** Expression of osteoclast markers was evaluated by western blotting. **F** TRAP staining results in each group. Experiments were performed for at least 3 times. Data are presented as mean ± SD. *P < 0.05, **P < 0.01 and ***P < 0.001

We then explored the function of miR-214-5p in the homeostasis of osteoclast. RAW264.7 cells were treated with miR-214-5p inhibitor or simultaneously with miR-214-5p inhibitor and sh ITGA7. Consistent with above results, miR-214-5p suppression activated ITGA7 obviously and addition of sh ITGA7 maintained ITGA7 expression at original level (Fig. 5A, B). The capability of proliferation and apoptosis was then examined by CCK-8 assay and flow cytometry, respectively. The results presented that block of miR-214-5p suppressed osteoclast proliferation and promoted apoptosis. While these changes could be fully reversed by ITGA7 knockdown (Fig. 5C, D). The expression of osteoclastogenesis-related markers in different group further corroborated this conclusion. The expression of multiple biomarkers, including TRAP, NFATc1,V-ATPase-d2 and CTSK were repressed by miR-214-5p inhibitor and recovered by sh ITGA7 (Fig. 5E). Correspondingly, the amount of TRAP positive cells also decreased in RANKL + miR-214-5p inhibitor group and was comparable to RANKL group after addition of sh ITGA7. Besides, we also examined the roles of miR-214-5p and ITGA7 in osteoblast. MiR-214-5p silencing has been shown to increase ITGA7 expression (Additional file 1: Fig. S1A-B). MiR-214-5p downregulation promoted proliferation and inhibited apoptosis in osteogenic-induced BMSCs, but ITGA7 downregulation reversed this effect (Additional file 1: Fig. S1C, D). MiR-214-5p deregulation facilitated osteogenic differentiation of BMSCs, but ITGA7 deregulation reversed this effect (Additional file 1: Fig. S1E). Collectively, miR-214-5p modulated bone homeostasis by regulating ITGA7.

### Block of miR-214-5p alleviates OVX-induced osteoporosis in animal model

**Fig. 6:**
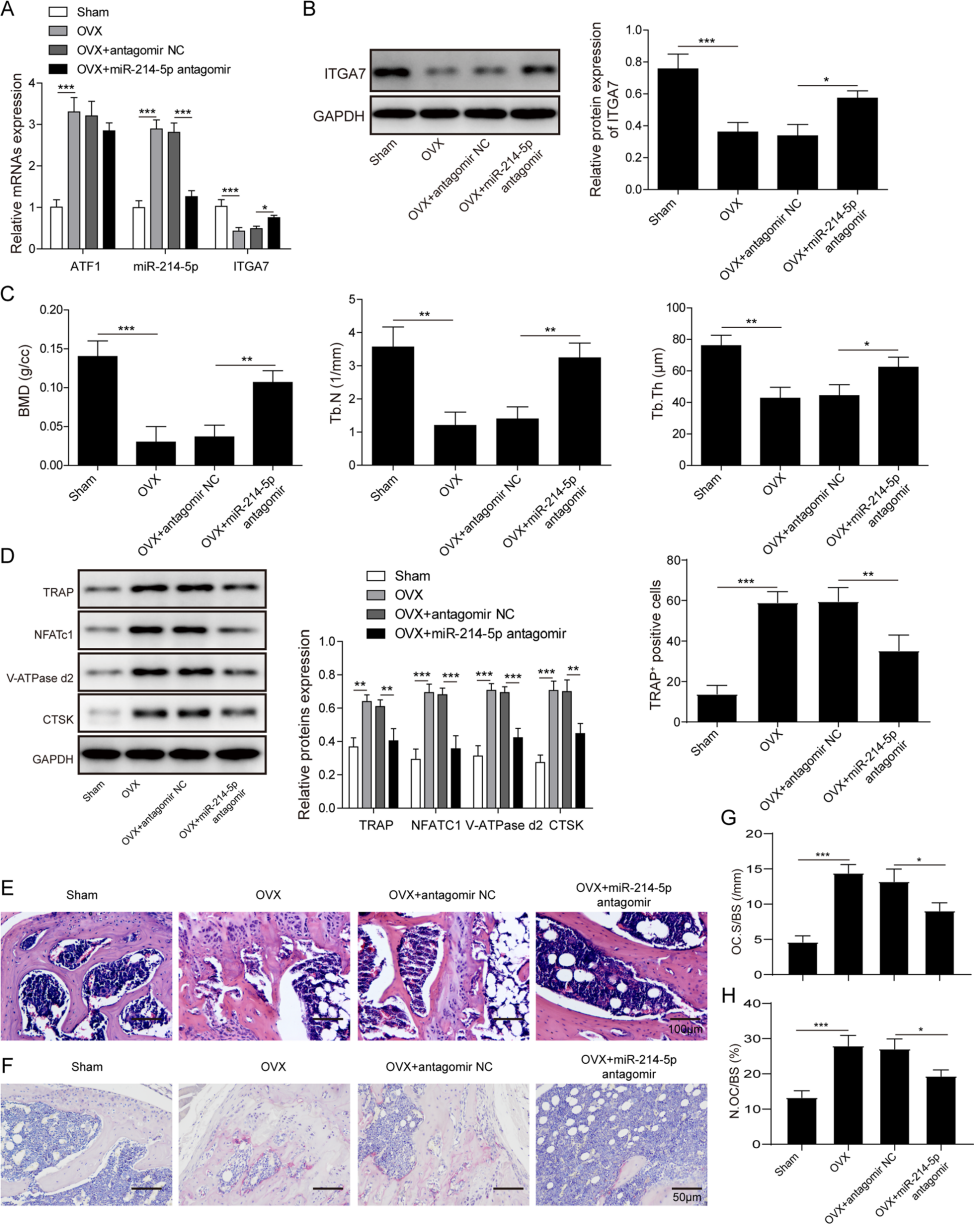
MiR-214-5p inhibition prevents OVX-induced osteoporosis. OVX or sham-operated mice were injected with miR-214-5p antagomir/antagomir NC. **A** Expression of miR-214-5p and ITGA7 was examined by qRT-PCR. **B** Protein level of ITGA7 was examined by western blotting. **C** Bone mineral density (BMD, g/cc), trabecular number (Tb.N, 1/mm) and trabecular thickness (Tb.Th, μm) of each sample were investigated by Micro CT. **D** Expression of osteoclast markers was evaluated by western blotting. **E**, **F** Bone tissue sections were exposed to H&E and TRAP staining. **G**, **H** Number of osteoclasts per bone surface (N.Oc/BS) and osteoclast surface per bone surface (Oc.S/BS) of mice obtained. N = 6. Data are presented as mean ± SD. *P < 0.05, **P < 0.01 and ***P < 0.001

We further verified in vitro results in mouse OVX model. Bone marrow cells from mouse backbone were collected for detection. ATF1 and miR-214-5p were upregulated at mRNA level after OVX treatment (Fig. 6A). ITGA7 was downregulated at both mRNA and protein level. Moreover, downregulation of miR-214-5p promoted ITGA7 expression in vivo (Fig. 6A-B). Micro-CT presented significant bone loss in OVX group. In miR-214-5p antagomir treated group, bone parameters, such as BMD, Tb.N (1/mm) and Tb.Th (μM) presented an obvious increase than in antagomir NC group (Fig. 6C). The expression of TRAP, NFATc1,V-ATPase-d2 and CTSK also increased in OVX group and decreased after treatment with miR-214-5p antagomir (Fig. 6D). HE staining demonstrated that increased bone loss and greater changes in bone surface and volume occurred in mice after OVX. Knockdown of miR-214-5p reversed these effects (Fig. 6E). TRAP staining showed that TRAP-positive osteoclast increased within OVX group. MiR-214-5p knockdown helped maintain bone structure and reduced the number of mature osteoclasts (Fig. 6F). Compared with Sham, N.Oc/BS and Oc.S/BS levels were increased in osteoporosis model, while down-regulation of miR-214-5p decreased N.Oc/BS and Oc.S/BS (Fig. 6G, H). In summary, miR-214-5p knockdown prevented OVX-induced osteoporosis in the mouse model.

## Discussion

The balance of osteoblasts and osteoclasts is essential for the health status of bone volume, structure and morphology (Zhang and Sugawara 2012). Osteoporosis may occur if the bone absorption of osteoclast exceeds the compensation of osteoblast (Ouyang et al. 2014). Now osteoporosis has become one of the dominant disease that compromise life quality, especially in elderly women. The prevalence of osteoporosis is 19.2% in Chinese aged more than 50 years. And this number could be up to 32.1% in female aged > 50 (Yu and Xia 2019). Osteoporosis remains under-managed in China and exploration of related mechanisms is urgently needed. In this research, we manifested that ATF1 activated miR-214-5p transcriptionally, thereby inhibiting downstream target ITGA7 and finally facilitating osteoclast formation and accelerating bone loss. This novel pathway in osteoclastogenesis could provide potential candidates for osteoporosis therapy.

In hyalinizing clear cell carcinoma, ATF1 could fuse with EWSR1 and was associated with tumor development (Weinreb 2013). However, studies of ATF1 in osteoclast formation are limited. ATF1 is a novel transcription factor in osteoclastogenesis and knockdown of ATF1 suppressed cell differentiation toward osteoclast (Guo et al. 2019). But the specific signaling pathway has not been clearly elucidated. Our study reported miR-214-5p was the direct downstream target of ATF1 in RAW264.7 cells and identified the target for the first time, which was a good supplement for the function of ATF1.

Possessing critical functions in the regulation of multiple biological process, miRNA was also reported playing essential roles in bone homeostasis. Guo et al. showed that MiRNA-218 contributed protective effect on periodontitis by direct targeting matrix metalloproteinase-9 (Mmp9) (Guo et al. 2019). Chen et al. revealed miR-195a could bind to circRNA 28,313 and CSF1 directly and regulate osteoclast differentiation (Chen et al. 2019). MiR-214-5p was previously revealed to exacerbate osteoporosis by affecting adipogenic differentiation of BMSCs (Qiu et al. 2018). However, no direct evidence showed miR-214-5p was directly associated with osteoclast differentiation. As a member of integrin family, ITGA7 was greatly inhibited in ovariectomy (OVX) mice. Nonetheless, interaction of miR-214-5p and ITGA7 was not mentioned previously (Yao et al. 1996). Our data exhibited that miR-214-5p directly bound to ITGA7. Downregulation of miR-214-5p activated ITGA7 and prevented osteoclast formation both in cells and in animal models.

## Conclusions

In summary, our research demonstrated ATF1 could directly activate miR-214-5p transcriptionally and further suppress ITGA7 expression. This pathway could regulate osteoclast formation in RANKL-induced cells and OVX mouse model. Therefore, this study contributes a novel pathway, ATF1/miR-214-5p/ITGA7 axis, involved in the regulation of bone resorption, which might provide a new option for the treatment of human osteoporosis.

## Supplementary Information

**Additional file 1: Figure S1.** Down-regulation of miR-214-5p promotes osteogenesis in BMSCs cells, but up-regulation of ITGA7 reverses the impact of miR-214-5p in vitro. BMSCs were treated with miR-214 inhibitor or inhibitor NC with or without sh ITGA7 or sh NC. Cells of control group were treated with saline. A. Expression of miR-214-5p and ITGA7 was examined by qRT-PCR. B. Protein level of ITGA7 was examined by western blotting. C. Cell viability was evaluated by CCK-8 assay in each group. D. Cell apoptosis was assessed by flow cytometry. E. Oesteogenic differentiation was detected by Alizarin red staining. Scale bar, 20 μm. Experiments were performed for at least 3 times. Data are presented as mean ± SD. *P < 0.05, **P < 0.01 and ***P < 0.001.

**Additional file 2: Figure S2.** ATF1, miR-214-5p are up-regulated, while ITGA7 is down-regulated in primary osteoclasts. A. Expression of osteoclast markers examined by western blotting. B. TRAP staining results in control and primary osteoclasts group. C. Expression of ATF1, miR-214-5p and ITGA7 examined by qRT-PCR. D. Protein expression of ITGA7 examined by western blotting. Experiments were performed for at least 3 times. Data are shown as mean ± SD. *P < 0.05, **P < 0.01 and ***P < 0.001.

## Data Availability

All data generated or analyzed during this study are included in this article. The datasets used and/or analyzed during the current study are available from the corresponding author on reasonable request.

## Abbreviations

ATF1: Activating transcription factor 1
ITGA7: Integrin alpha-7
OVX: Ovariectomized
qRT-PCR: Quantitative real-time PCR
IHC: Immunohistochemistry
ChIP: Chromatin Immunoprecipitation
DMEM: Dulbecco Modified Eagle Medium
FBS: Fetal bovine serum
shRNA: Short hairpin RNA
WT: Wild type
MUT: Mutated-type
TBST: Tris Buffered Saline Tween
SD: Standard deviation
ANOVA: Analysis of variance
GAPDH: Glyceraldehyde-3-phosphate dehydrogenase
RIPA: Radio immunoprecipitation
IgG: Immunoglobulin G
HRP: Horseradish peroxidase
NC: Negative control
miRNA: MicroRNA
3′UTR: 3′-Untranslated region
BMD: Bone mineral density
Tb.N: Trabecular number
Tb.Th: Trabecular thickness
TRAP: Tartrate-resistant acid phosphatase
RANKL: Receptor activator of nuclear factor-κB ligand
CSF1: Colony-stimulating factor
MOI: Multiplicity of infection
oe: Overexpression
