# Supplementary figures and images for: ATF1/miR-214-5p/ITGA7 axis promotes osteoclastogenesis to alter OVX-induced bone absorption
Source: Mol Med. 2022 May 14;28:56. doi: 10.1186/s10020-022-00476-7 (PMC9107670; doi:10.1186/s10020-022-00476-7)

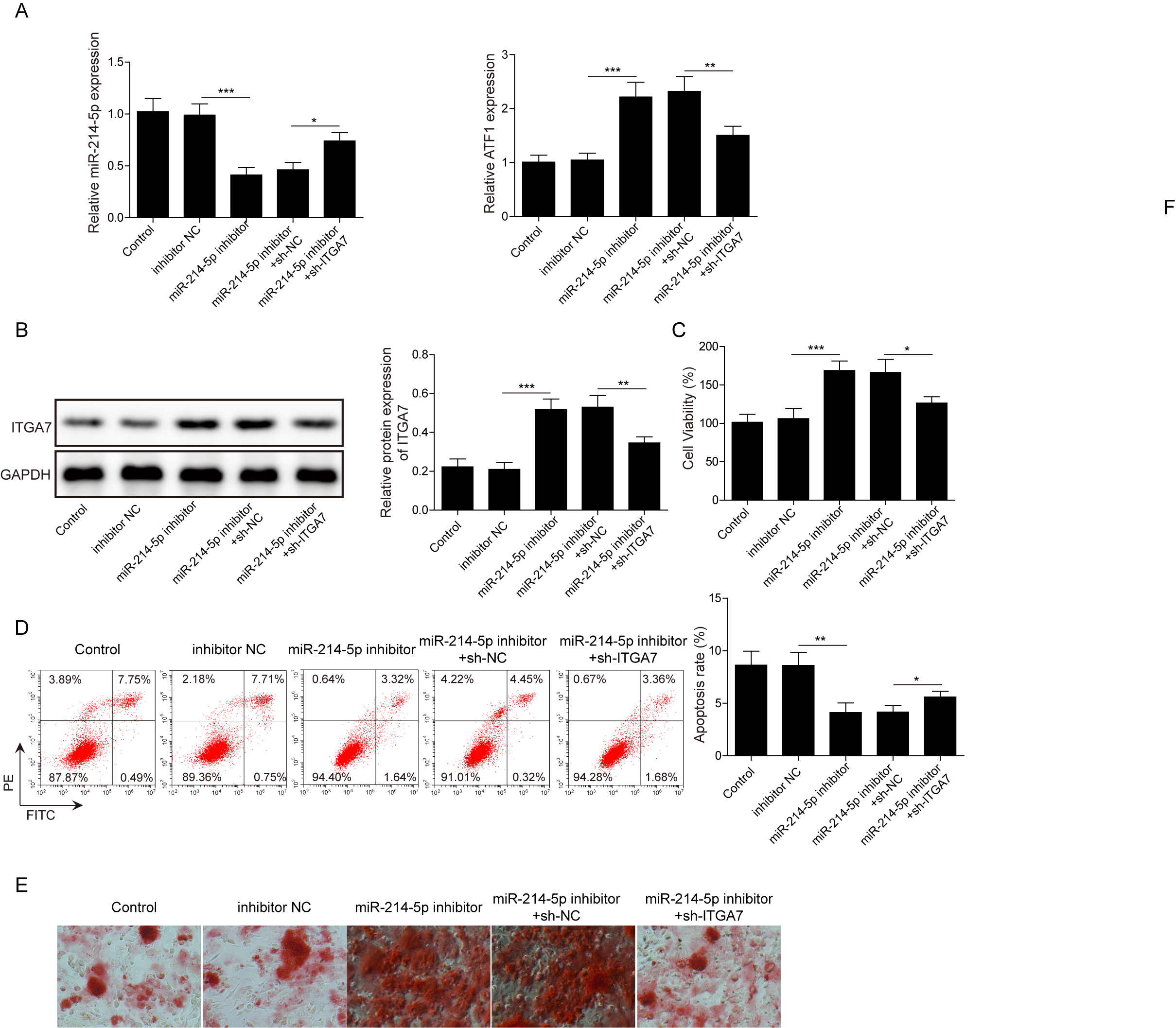

Supplement: Supplementary file 1 — Additional file 1: Figure S1. Down-regulation of miR-214-5p promotes osteogenesis in BMSCs cells, but up-regulation of ITGA7 reverses the impact of miR-214-5p in vitro. BMSCs were treated with miR-214 inhibitor or inhibitor NC with or without sh ITGA7 or sh NC. Cells of control group were treated with saline. A. Expression of miR-214-5p and ITGA7 was examined by qRT-PCR. B. Protein level of ITGA7 was examined by western blotting. C. Cell viability was evaluated by CCK-8 assay in each group. D. Cell apoptosis was assessed by flow cytometry. E. Oesteogenic differentiation was detected by Alizarin red staining. Scale bar, 20 μm. Experiments were performed for at least 3 times. Data are presented as mean ± SD. *P < 0.05, **P < 0.01 and ***P < 0.001. [file 10020_2022_476_MOESM1_ESM.tif]

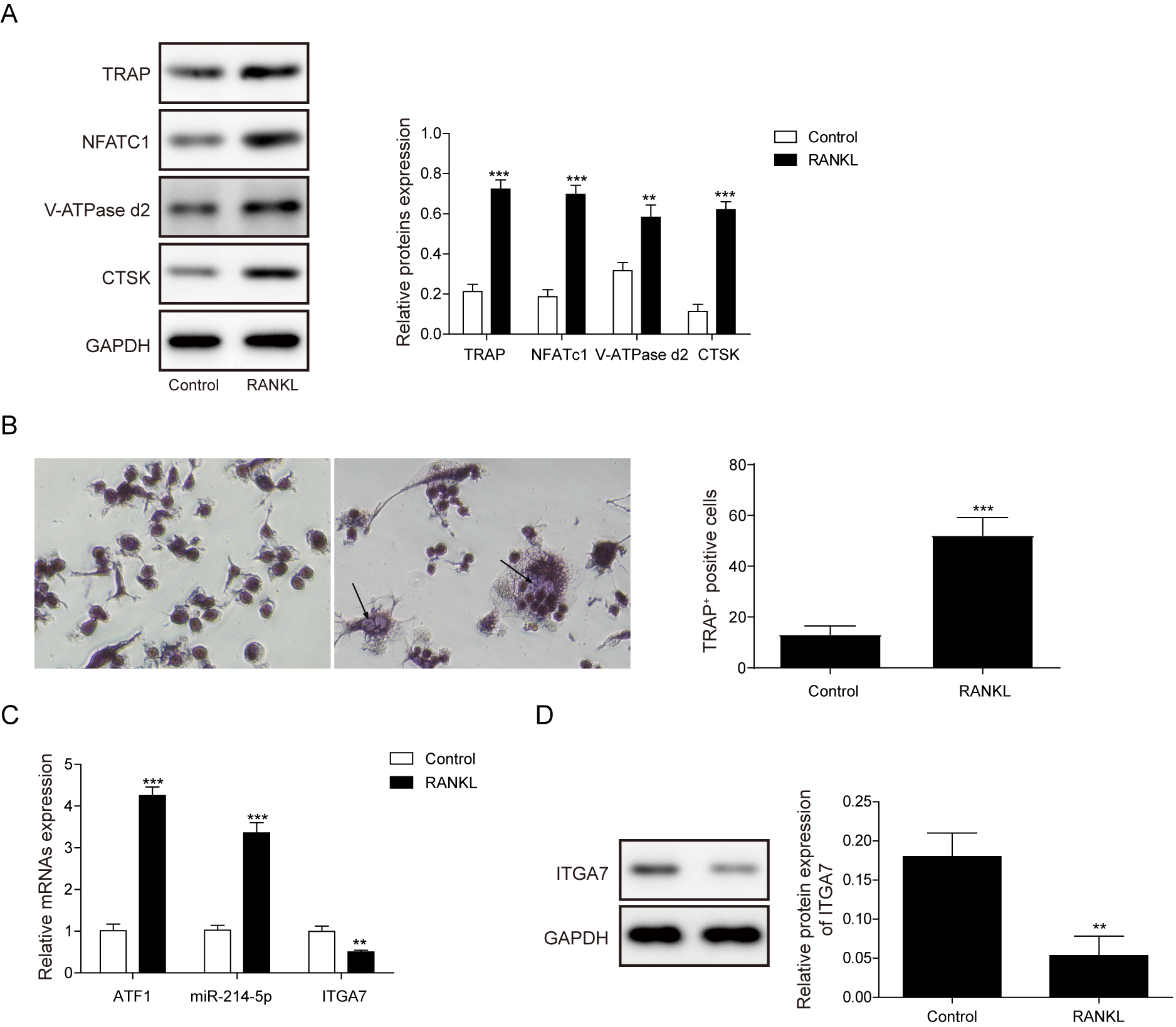

Supplement: Supplementary file 2 — Additional file 2: Figure S2. ATF1, miR-214-5p are up-regulated, while ITGA7 is down-regulated in primary osteoclasts. A. Expression of osteoclast markers examined by western blotting. B. TRAP staining results in control and primary osteoclasts group. C. Expression of ATF1, miR-214-5p and ITGA7 examined by qRT-PCR. D. Protein expression of ITGA7 examined by western blotting. Experiments were performed for at least 3 times. Data are shown as mean ± SD. *P < 0.05, **P < 0.01 and ***P < 0.001. [file 10020_2022_476_MOESM2_ESM.tif]
